# Circular RNA circPITX1 knockdown inhibits glycolysis to enhance radiosensitivity of glioma cells by miR-329-3p/NEK2 axis

**DOI:** 10.1186/s12935-020-01169-z

**Published:** 2020-03-14

**Authors:** Yongchang Guan, Zhi Cao, Jinghua Du, Tao Liu, Tingzhong Wang

**Affiliations:** Department of Neurosurgery, The Fourth Affiliated Hospital of China Medical University, No. 4 Chongshan Road, Huanggu District, Shenyang, 110000 Liaoning China

**Keywords:** Glioma, circPITX1, miR-329-3p, NEK2, Glycolysis, Radiosensitivity, Radioresistance

## Abstract

**Background:**

Numerous circular RNAs (circRNAs) have been recognized as vital modulators of human malignancies, including glioma. Whereas, the functional role of circRNA Pituitary Homeo Box 1 (circPITX1) in the radioresistance of glioma cells remains largely uncertain.

**Methods:**

Quantitative real-time PCR (qRT-PCR) or western blot analysis was employed to examine the expression of circPITX1, microRNA (miR)-329-3p and NIMA-related kinase 2 (NEK2). 3-(4, 5-dimethylthiazol-2-y1)-2, 5-diphenyl tetrazolium bromide (MTT) assay was used to determine cell viability. Glycolysis was assessed by commercial kits and western blot analysis. Colony formation assay was conducted to analyze cell survival and clonogenicity capacity. The relationship among circPITX1, miR-329-3p and NEK2 was confirmed via dual-luciferase reporter assay. The in vivo function of circPITX1 was evaluated by tumor xenograft assay.

**Results:**

Expression of circPITX1 and NEK2 was up-regulated in glioma tissues and cells, while miR-329-3p exhibited reverse trend. CircPITX1 knockdown repressed viability, glycolysis and colony formation, but promoted radiosensitivity of glioma cells, as well as inhibited tumor growth in vivo. MiR-329-3p was a target miRNA of circPITX1 and miR-329-3p deficiency reversed knockdown of circPITX1-mediated glycolysis inhibition and radioresistance reduction. MiR-329-3p exerted inhibitory effects on glycolysis and radioresistance of glioma cells by targeting NEK2. CircPITX1 facilitated NEK2 expression by sponging miR-329-3p. Glycolytic inhibitor 2-deoxy-d-glucose (2-DG) disposition weakened the promoted impact on glycolysis caused by circPITX1.

**Conclusion:**

CircPITX1 knockdown reduced glycolysis to contribute to radiosensitivity in glioma through miR-329-3p/NEK2 axis, providing a possible mechanism of circPITX1 in the development of glioma.

## Highlights

1. CircPITX1 and NEK2 levels are up-regulated in glioma, while miR-329-3p expression is decreased.
2. CircPITX1 knockdown inhibits cell viability, glycolysis, radioresistance, cell survival and clonogenicity capacity of glioma cells in vitro and blocks tumor growth in vivo.
3. CircPITX1 up-regulates NEK2 by targeting miR-329-3p.
4. CircPITX1 knockdown represses glycolysis to enhance radiosensitivity in glioma.

## Background

In adults, glioma and CNS lymphomas rank as the two most prevailing brain tumors. Currently, the therapy drugs of glioma mainly include steroid, anticonvulsant and agents that alleviate worry and anxiety [1]. Additionally, surgical resection and radiotherapy are also optional treatment approaches, in which radiotherapy is an ancillary treatment method, for it is advantageous to the survival of glioma patients. However, the outcome of radiotherapy often discounts as a result of radioresistance in glioma [2]. Thus, it is necessary to reduce the radioresistance of glioma cells.

For cancer cells, and even various normal cells often display high rates of glycolysis, no matter the oxygen is enough or not, namely the “Warburg effect” [3]. Glycolysis indicates a process of transformation from glucose to pyruvate followed by lactate production, providing cellular energy and involving in macromolecular biosynthesis, which has potential to be therapeutic target for human cancers [4]. The glycolytic process is usually accompanied by glucose uptake and lactate production, as well as ATP generation, a vital determination factor of cellular chemoresistance [4]. Hypoxia inducible factor (HIF)-1α is recognized as a vital modulator of the glycolytic pathway activity [5]. Hexokinases I and II (HK1 and HK2) were manifested to affect the glucose metabolism and tumorigenicity in the development of colorectal cancer and melanoma [6]. Lactate dehydrogenase A (LDH-A) is a crucial enzyme during glycolytic process [7]. A former literature reports that dichloroacetate could elevate the radiosensitivity of glioblastoma cells through regulating the glycolysis [8]. Therefore, exploring the glycolysis through detecting above related genes in glioma is of great significance.

Circular RNAs (circRNAs) are a category of endogenous RNA molecules, characterized by the covalently closed loop structure, with abundant expression in mammalian cells [9]. CircRNA functions through a variety of ways, such as serving as competing endogenous RNA (ceRNA) or transcriptional regulator combining with RNA binding proteins and being translated to proteins [10]. Multiple circRNAs are identified as cancer-associated in glioma. CircRNA circHIPK3 could facilitate glioma progression through sponging miR-654 to up-regulate IGF2BP3, exhibiting as the elevated impact on cell proliferation and invasion as well as tumor propagation [11]. Hsa-circ-0014359 was suggested to exert oncogenic role in glioma progression via the regulation of miR-153/PI3K signaling [12]. CircCPA4 functions as a prognostic element and promotes malignant behaviors in glioma [13]. CircRNA Pituitary Homeo Box 1 (circPITX1) located in chr5: 134363423-134365011, also named as hsa-circ-0074026, is found to be up-regulated in glioma tissues relative to noncancerous controls through high-throughput circRNA sequencing [14]. CircPITX1, produced via splicing of PITX1, could aggravate glioma progression by miR-1304/ERBB4 axis, acting as an indicator of poor prognosis of patients with glioma [15]. Nevertheless, the novel mechanistic pathway of circPITX1 in the development and radioresistance of glioma needs to be explored.

MicroRNAs (miRNAs), short noncoding RNAs, play important part in glioma tumorigenesis and progression [16], and are identified as valued biomarkers for glioma prognosis in clinical, including miR-15b, miR-21, miR-148a, miR-196, miR-210, miR-221, miR-106a, and miR-124 [17]. Mapped on 14q32.31, miR-329-3p exerts a tumor-suppressive role in osteosarcoma, hepatocellular carcinoma, non-small cell lung cancer and cervical cancer, implicated with the proliferation and metastasis of cancer cells [18]. MiR-329 is decreased in glioblastoma tissue samples, and could act as prognostic indicator for the outcome of patients with glioblastoma in clinic [19]. Regrettably, the precise functional role of miR-329-3p in glioma is not fully expounded.

As a member of the NIMA-related kinase family, NIMA-related kinase 2 (NEK2) is a conserved centrosome kinase, with ectopic expression in different kinds of human cancers [20]. In prostate cancer, overexpression of NEK2 contributes to the proliferation and tumorigenicity of LNCaP cells, and is correlated to the poor prognosis of patients with prostate cancer [21]. In glioma, NEK2 has been suggested to be correlated with malignancy and the poor overall survival of patients with glioma [22]. Here, we intended to clarify whether NEK2 was involved in circPITX1-mediated glioma progression.

In this study, we evaluated the expression pattern of circPITX1 and its impact on glycolysis and radioresistance of glioma U251 and LN229 cells. We also explored the potential action mechanism.

## Materials and methods

### Collection of clinical tissues

32 glioma tissues (derived from glioma patients with histological diagnosis) and 32 normal brain tissues (derived from patients with spontaneous intracerebral hemorrhage and matching age and gender) were collected at the Fourth Affiliated Hospital of China Medical University. Resected tissues were preserved at − 80 °C. Above participators signed informed consent and they hadn’t received any other anti-tumor treatment. Our investigation was conducted with the approval of the Ethics Committee of the Fourth Affiliated Hospital of China Medical University.

### Cell culture

Normal human astrocyte (NHA, BNCC341796) and human glioma U251 cells (BNCC100497) were commercially obtained from BeNa Culture Collection (Beijing, China). And human glioma LN229 cells (CRL-2611) were purchased from American Type Culture Collection (ATCC, Manassas, VA, USA). Cell culture was conducted in a 5% CO_2_ humidity incubator at 37 °C utilizing Roswell Park Memorial Institute 1640 Medium (RPMI1640; Invitrogen, Carlsbad, CA, USA) mixed with 10% fetal bovine serum (Invitrogen) and 1% penicillin/streptomycin (Invitrogen).

### Quantitative real-time PCR (qRT-PCR)

For RNA extraction, TRIzol Reagent (Invitrogen) was used. Then, the complementary DNA (cDNA) was synthesized with 1 μg RNA as template using M-MLV Reverse Transcriptase (Invitrogen) or miScript Reverse Transcription Kit (QIAGEN, Hilden, Germany). qRT-PCR was implemented utilizing QuantiTect SYBR Green PCR Kit (Qiagen) or all-in-one miRNA RT-qPCR Detection Kit (GeneCopoeia, Guangzhou, China). The relative expression of circPITX1, PITX1 mRNA, miR-329-3p and NEK2 was calculated through 2^−∆∆Ct^ method, with glyceraldehyde-3-phosphate dehydrogenase (GAPDH; for circPITX1, PITX1 mRNA and NEK2) or U6 (for miR-329-3p) as internal reference. Primer sequences were: circPITX1: 5′-GCGTCCCTGTGTATGTTGGA-3′ (sense) and 5′-GTCTGTCTTAAAGCGACAGCG-3′ (anti-sense); PITX1: 5′-GTACGCACTTCACAAGCCAGCA-3′ (sense) and 5′-GCTCGGTGAGGTTGGTCCACA-3′ (anti-sense); miR-329-3p: 5′-GTGGAACAGACCTGGTAAAC-3′ (sense) and 5′-CAAGTGCGAGTCGTGCAGT-3′ (anti-sense); NEK2: 5′-TGCTTCGTGAACTGAAACATCC-3′ (sense) and 5′-CCAGAGTCAACTGAGTCATCACT-3′ (anti-sense); GAPDH: 5′-TCCTGCACCACCAACTGTTT-3′ (sense) and 5′-GGATGATGTTCTGGTGGGCA-3′ (anti-sense); U6: 5′-GCTTCGGCAGCACATATACTAAAAT-3′ (sense) and 5′-CGCTTCACGAATTTGCGTGTCAT-3′ (anti-sense).

### RNase R treatment

To determine the stabilization of circPITX1, 10 μg RNA extracted from U251 and LN229 cells was incubated with RNase R (4 U/μg; Epicentre Biotechnologies, Madison, WI, USA) or not at 37 °C for 1 h. Later, the relative expression of circPITX1 and PITX1 mRNA was examined by qRT-PCR.

### Cell transfection

Until 60% of coverage, glioma U251 and LN229 cells were transfected with Lipofectamine 3000 (Invitrogen). For silencing circPITX1, small interfering RNA (siRNA) of circPITX1 (si-circPITX1) and the control (si-NC) were synthesized by KeyGEN Biotech (Jiangsu, China). For up-regulating circPITX1 or NEK2, the sequence was cloned into pcDNA3.1 vector (Promega, Southampton, UK), namely pcDNA-circPITX1 or pcDNA-NEK2, generating in Hanbio Biotechnology Co., ltd (Shanghai, China), with pcDNA-NC as control. MiR-329-3p inhibitors (anti-miR-329-3p), miR-329-3p mimics (miR-329-3p) and their paired control (anti-miR-NC and miR-NC) were obtained from GeneCopoeia.

### X-ray irradiation treatment

Transfected U251 and LN229 cells were inoculated into 24-well plates overnight, and then treated with X-ray irradiation at different doses (0 Gy, 2 Gy, 4 Gy, 6 Gy and 8 Gy) for the determination of cell survival rate.

### 3-(4,5-Dimethylthiazol-2-y1)-2,5-diphenyl tetrazolium bromide (MTT) assay

During exponential growth phase, transfected U251 and LN229 cells were seeded in 96-well plates within RPMI1640 with 10% fetal bovine serum overnight. Next, 20 μL MTT (0.5 mg/mL, Invitrogen) was instilled into each well at 0 h, 24 h, 48 h and 72 h, respectively. 4 h later, culture medium was removed carefully. Then dimethyl sulfoxide (DMSO, Invitrogen) was dropped into each well. Absorbance at 490 nm was recorded by a microplate reader (BioTek Instruments Inc., Winooski, VT, USA).

### Detection of glucose consumption, lactate production and ATP level

To evaluate the glycolysis process, the glucose consumption, lactate production and ATP level were examined exploiting Glucose Assay Kit (ab65333; Abcam, Shanghai, China), Lactate Assay Kit (ab83429; Abcam) and ATP Assay Kit (ab83355; Abcam) severally, in conformity to the manufacturer’s instructions.

### Western blot

Protein sample preparation was implemented via Radio-Immunoprecipitation Assay (Vazyme, Nanjing, China). Following concentration determination using BCA Protein Quantification Kit (Vazyme), 40 µg protein samples were subjected to sodium dodecyl sulfate polyacrylamide gel electrophoresis, then transferred to polyvinylidene fluoride membranes (Thermo Fisher Scientific Inc., Waltham, MA, USA). After blockage in non-fat milk, the membranes were incubated into the diluted primary antibodies, including anti-hypoxia inducible factor-1 (anti-HIF-1α; 1:2000; ab51608; Abcam), anti-HK2 (1:2000; ab209847; Abcam), anti-LDH-A (1:2000; ab101562; Abcam), anti-NEK2 (1:2000; ab109283; Abcam) and anti-GAPDH (1:2000; ab181602; Abcam) at 4 °C overnight, then incubated in diluted secondary antibody (1:5000; ab205718; Abcam) at room temperature for 1 h. The visualization of protein blot was achieved utilizing an enhanced chemiluminescence detection kit (Thermo Fisher Scientific Inc.). Besides, the band intensity was tested using Image J software (NIH, Bethesda, MD, USA).

### Colony formation assay

The current assay was employed to monitor the cell survival rate and clonogenicity ability of glioma U251 and LN229 cells. In brief, 200 cells per well were seeded into 6-well plates with X-ray irradiation treatment (0 Gy, 2 Gy, 4 Gy, 6 Gy and 8 Gy) or not. At 14 days post incubation, generated colonies (indicating cell clumps with exceeding 50 cells) were fixed and stained with crystal violet at room temperature for 1 h. The colonies were photographed and counted. The survival rate was computed based on the formula: amount of colonies/(amount of inoculated cells × plating efficiency), and the survival curve was plotted. The colony formation rate was calculated by the analyzation of the percentage of cells which formed colony relative to the seeded cells.

### Dual-luciferase reporter assay

Potential targets of circPITX1 or miR-329-3p were searched by StarBase 3.0 (http://starbase.sysu.edu.cn/). To construct luciferase reporters, the sequence of circPITX1 or NEK2 3′ untranslated region (3′UTR) containing the forecasted binding sites or mutant binding sites was inserted into pGL4 vector (Promega Corp., Madison, WI, USA), namely circPITX1-WT, circPITX1-MUT, NEK2 3′UTR-WT and NEK2 3′UTR-MUT. U251 and LN229 cells were cotransfected with luciferase reporter and miR-329-3p or miR-NC using Lipofectamine 3000. After 48 h, the luciferase activity was examined via a Dual Luciferase Reporter Assay Kit (Vazyme) referring to the protocols supplied by the manufacturer.

### Tumor xenograft assay

This experiment was endorsed by the Animal Ethics Committee of the Fourth Affiliated Hospital of China Medical University. 12 BALB/c nude mice (male, 5-week old) were provided by Charles River (Beijing, China) and evenly divided into two groups. The short hairpin RNA (shRNA) against circPITX1 (sh-circPITX1) and its control sh-NC were synthesized by Genomeditech (Shanghai, China). 1 × 10^6^ LN229 cells stably expressing sh-circPITX1 or sh-NC were subcutaneously injected into the right flank of each mouse. 1 week later, the volume of tumor was recorded once a week, with the formula: 0.5 × length × width^2^. 5 weeks later, the formed tumor in nude mice was excised, photographed, weighed, and then collected for qRT-PCR assay or western blot analysis.

### Statistical analysis

All data were from at least 3 independent repetition experiments and processed exploiting GraphPad Prism 7 (GraphPad Inc., La Jolla, CA, USA), then presented as the mean ± standard deviation. Difference comparison depended on Student’s *t*-test and one-way analysis of variance followed by Tukey’s test. The correlation among the expression of circPITX1, miR-329-3p and NEK2 in glioma tissues was analyzed by Pearson correlation analysis. *P *< 0.05 indicated a significant difference.

## Results

### Up-regulation of circPITX1 in glioma tissues and cells

**Fig. 1:**
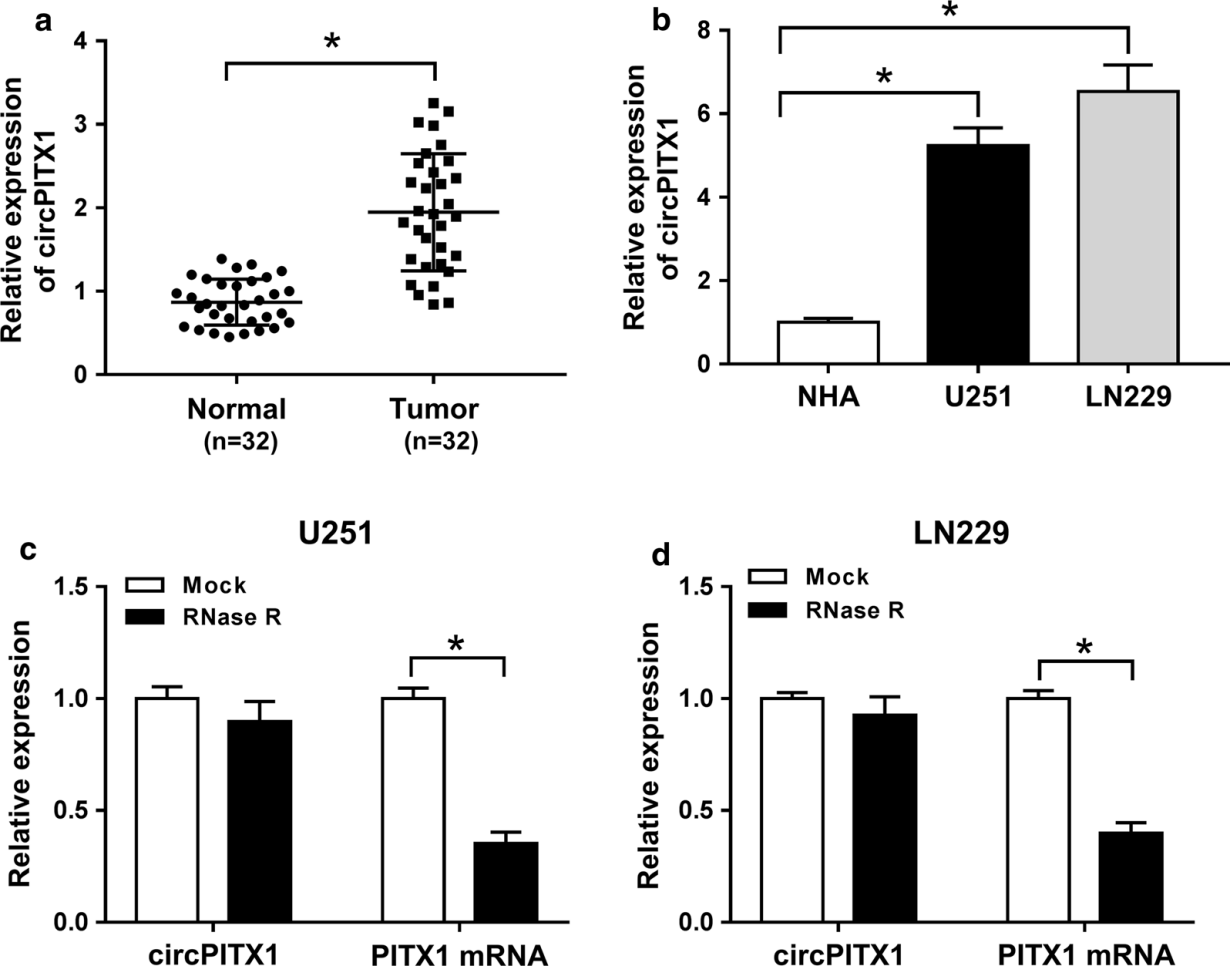
Up-regulation of circPITX1 in glioma tissues and cells. **a**, **b** QRT-PCR assay for the relative expression of circPITX1 in glioma tissues and normal tissues, as well as in glioma U251 and LN229 cells and NHA. **c**, **d** QRT-PCR assay for the relative expression of circPITX1 and PITX1 mRNA in U251 and LN229 cells disposed with RNase R or not (Mock). *P < 0.05

The qRT-PCR assay was applied to analyze the relative expression of circPITX1. As shown in Fig. 1a, b, circPITX1 abundance was higher in glioma tissues and glioma U251 and LN229 cells than that in normal tissues and NHA, respectively. To validate the stability of circPITX1, RNase R was exploited. The expression of PITX1 mRNA in U251 and LN229 cells was largely decreased after RNase R disposition, while circPITX1 was unaffected (Fig. 1c, d), suggesting that circPITX1 was more resistant to RNase R digestion [10]. In total, circPITX1 was a stable circRNA and might be associated with glioma progression.

### Impact of circPITX1 knockdown on glycolysis and radioresistance of glioma cells

**Fig. 2:**
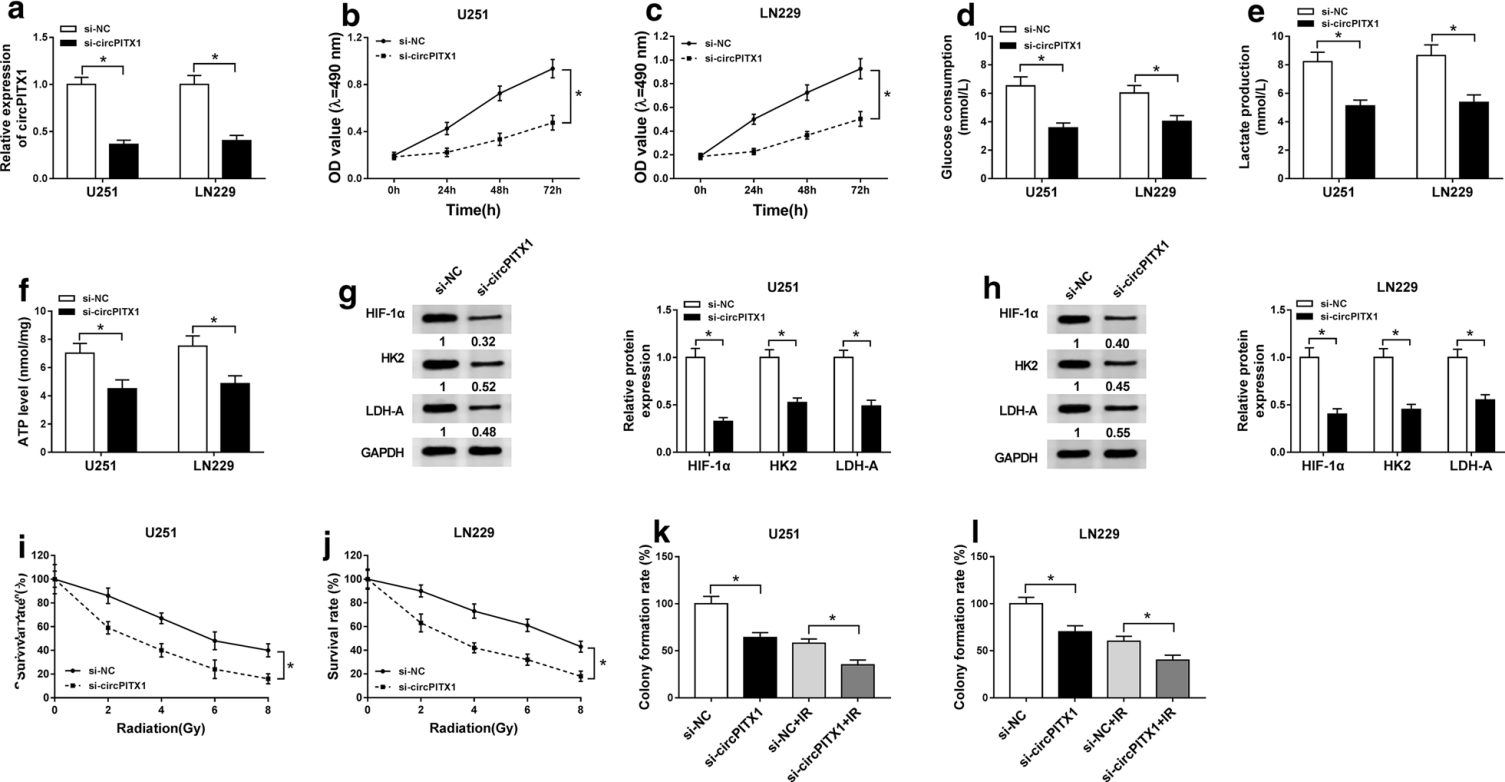
Impact of circPITX1 knockdown on glycolysis and radioresistance of glioma cells. **a**–**h** U251 and LN229 cells were transfected with si-circPITX1 or si-NC. **a** QRT-PCR assay for the relative expression of circPITX1 in transfected glioma cells. **b**, **c** MTT assay for the viability of transfected glioma cells. **d**–**f** The glucose consumption, lactate production and ATP level in transfected U251 and LN229 cells. **g**, **h** Western blot assay for the levels of HIF-1α, HK2 and LDH-A in transfected U251 and LN229 cells. **i**, **j** Colony formation assay for the survival rate of U251 and LN229 cells transfected with si-circPITX1 or si-NC under X-ray radiation treatment (0 Gy, 2 Gy, 4 Gy, 6 Gy and 8 Gy). **k**, **l** Colony formation assay for the colony formation rate of U251 and LN229 cells transfected with si-circPITX1 or si-NC treated with 6 Gy of X-rays or not. *P < 0.05

To clarify the biological role of circPITX1 in glioma progression, we knocked down circPITX1 by the transfection of si-circPITX1 into glioma cells, with si-NC as control. Through qRT-PCR assay, the knockdown efficiency was excellent, leading to about 60% reduction of circPITX1 expression in U251 and LN229 cells (Fig. 2a). MTT assay manifested that silence of circPITX1 greatly suppressed cell viability of U251 and LN229 cells (Fig. 2b, c). Glycolysis assay data showed that circPITX1 knockdown apparently inhibited glucose consumption, lactate production and ATP level in transfected U251 and LN229 cells (Fig. 2d–f). Following western blot analysis was performed to determine the levels of glycolysis-related factors (HIF-1α, HK2 and LDH-A) [23]. As illustrated in Fig. 2g, h, levels of HIF-1α, HK2 and LDH-A were decreased in si-circPITX1 treated U251 and LN229 cells. Besides, similar declined tendency was discovered in the survival rate of glioma cells with circPITX1 knockdown under radiation treatment (0 Gy, 2 Gy, 4 Gy, 6 Gy and 8 Gy) (Fig. 2i, j). Silence of circPITX1 could reduce the colony formation rate of transfected U251 and LN229 cells with or without radiation treatment (Fig. 2k, l). Altogether, circPITX1 knockdown hampered glycolysis and radioresistance of glioma cells.

### MiR-329-3p was a target of circPITX1

**Fig. 3:**
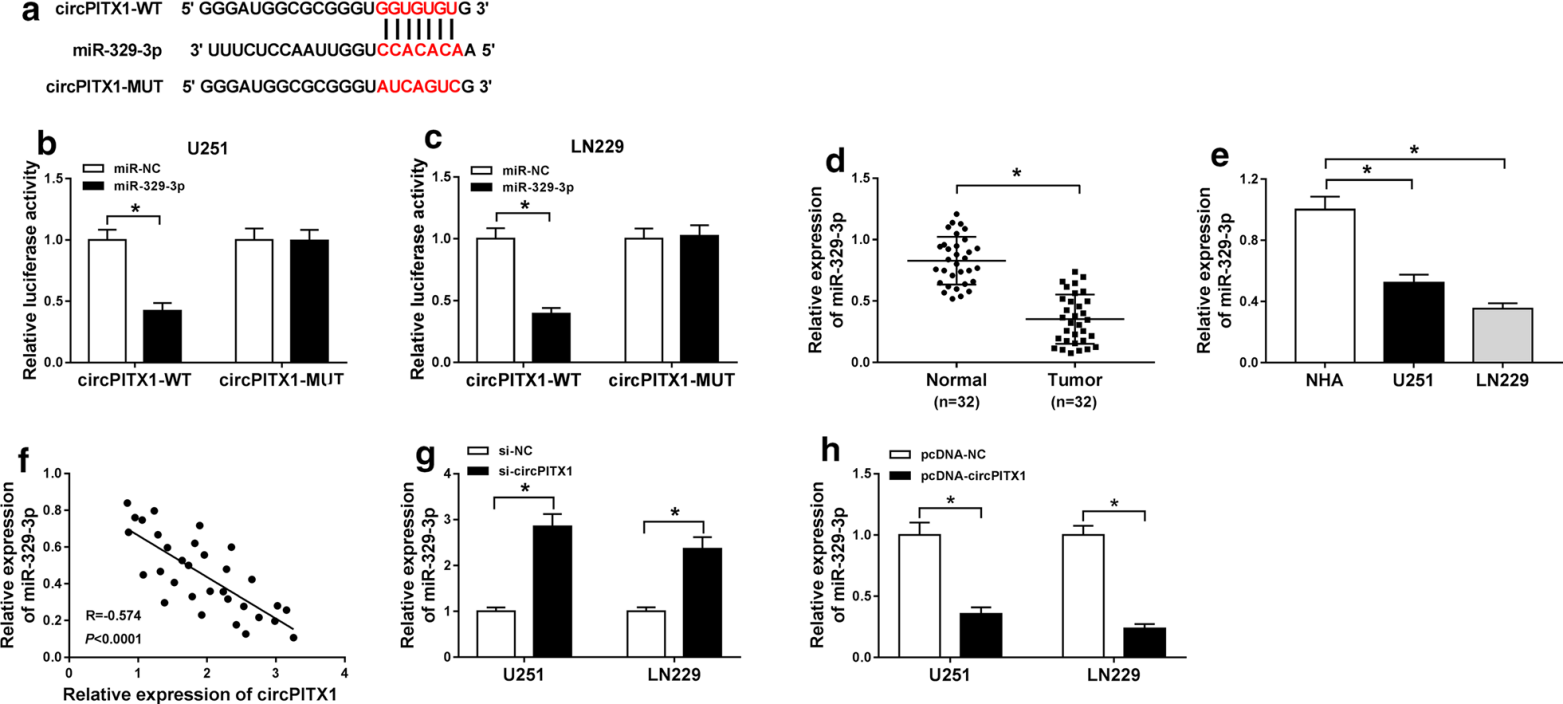
MiR-329-3p was a target of circPITX1. **a** The binding sites between circPITX1 and miR-329-3p, as well as the mutant. **b**, **c** Dual-luciferase reporter assay for the luciferase activity in glioma U251 and LN229 cells cotransfected with circPITX1-WT or circPITX1-MUT and miR-329-3p or miR-NC. **d**, **e** QRT-PCR assay for the relative expression of miR-329-3p in glioma tissues and normal tissues, as well as in glioma U251 and LN229 cells and NHA. **f** Pearson correlation analysis for the correlation between the expression of circPITX1 and miR-329-3p in glioma tissues. R = − 0.574, *P *< 0.0001. **g** QRT-PCR assay for the relative expression of miR-329-3p in U251 and LN229 cells transfected with si-circPITX1 or si-NC. **h** QRT-PCR assay for the relative expression of miR-329-3p in U251 and LN229 cells transfected with pcDNA-circPITX1 or pcDNA-NC. *P < 0.05

The action mechanism of circPITX1 in the cellular behaviors of glioma cells was the investigation focus hereinafter. As StarBase 3.0 predicted, there existed binding sites between circPITX1 and miR-329-3p (Fig. 3a). To further confirm the targeted relation, dual-luciferase reporter assay was conducted and revealed that introduction of miR-329-3p led to an about 60% decline of the luciferase activity in circPITX1-WT group, but it did not affect the luciferase activity when the seed sites were mutated in circPITX1-MUT group (Fig. 3b, c). Moreover, miR-329-3p expression was demonstrated to be lowered in glioma tissues and cells (NHA) in contrast to that in normal tissues and glioma U251 and LN229 cells, respectively (Fig. 3d, e). A negative correlation between the expression of circPITX1 and miR-329-3p in glioma tissues was validated by Pearson correlation analysis (Fig. 3f). We also observed that silence of circPITX1 caused 2.5-fold increase of miR-329-3p level, while up-regulation of circPITX1 constrained miR-329-3p enrichment, leading to approximately 70% reduction, with respect to corresponding controls (Fig. 3g, h). Above data proved that circPITX1 targeted miR-329-3p and inversely regulated miR-329-3p.

### CircPITX1 knockdown exerted inhibitory effects on the glycolysis and radioresistance of glioma cells by sponging miR-329-3p

**Fig. 4:**
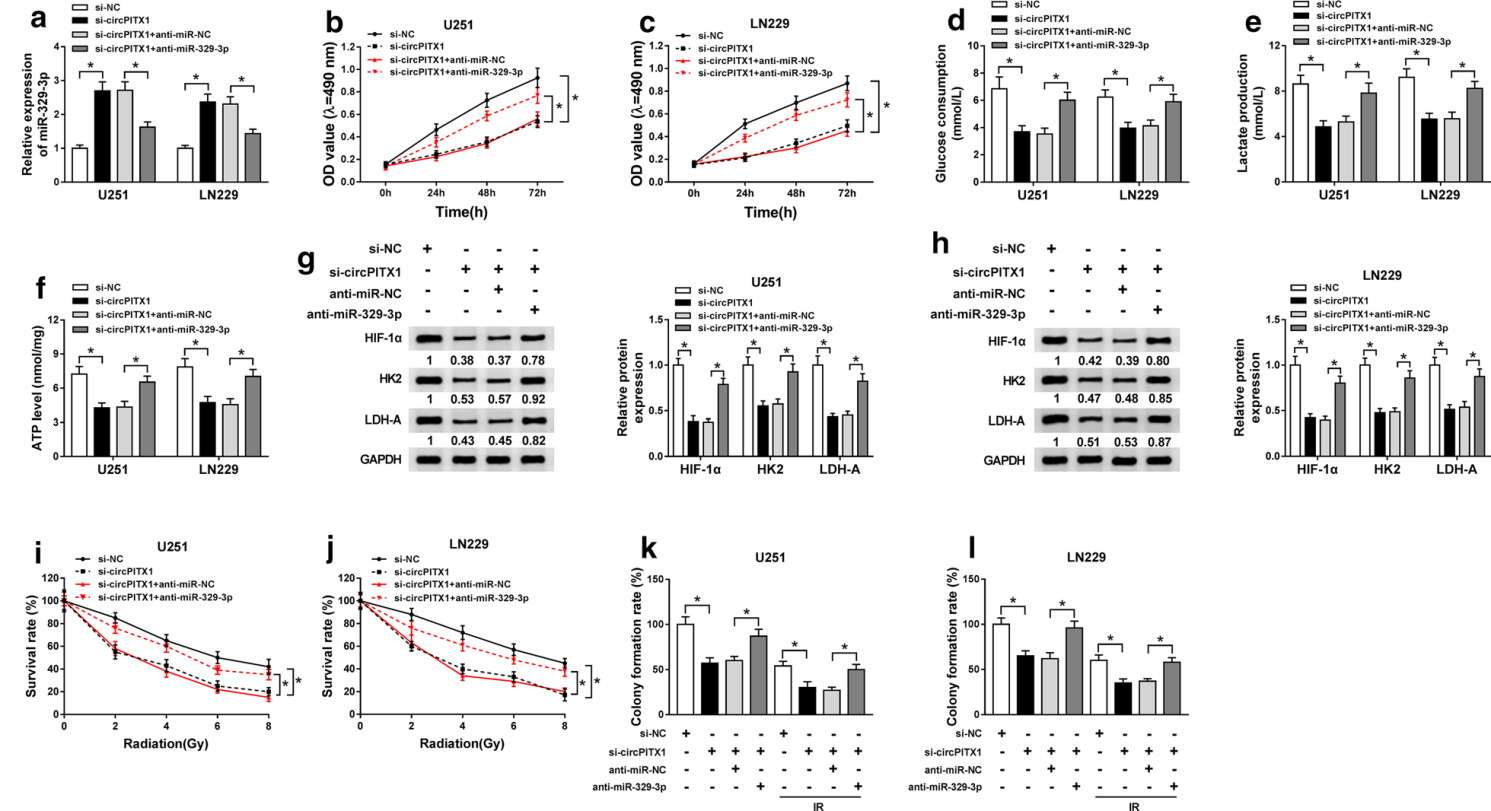
CircPITX1 knockdown exerted inhibitory effects on the glycolysis and radioresistance of glioma cells by sponging miR-329-3p. **a**–**h** U251 and LN229 cells were transfected with si-NC, si-circPITX1, si-circPITX1 + anti-miR-NC or si-circPITX1 + anti-miR-329-3p. **a** QRT-PCR assay for the relative expression of miR-329-3p in transfected glioma cells. **b**, **c** MTT assay for the viability of transfected glioma cells. **d**–**f** The glucose consumption, lactate production and ATP level in transfected U251 and LN229 cells. **g**, **h** Western blot assay for the levels of HIF-1α, HK2 and LDH-A in transfected U251 and LN229 cells. **i**, **j** Colony formation assay for the survival rate of U251 and LN229 cells transfected with si-NC, si-circPITX1, si-circPITX1 + anti-miR-NC or si-circPITX1 + anti-miR-329-3p under X-ray radiation treatment (0 Gy, 2 Gy, 4 Gy, 6 Gy and 8 Gy). **k**, **l** Colony formation assay for the colony formation rate of U251 and LN229 cells transfected with si-NC, si-circPITX1, si-circPITX1 + anti-miR-NC or si-circPITX1 + anti-miR-329-3p treated with 6 Gy of X-rays or not. *P < 0.05

Having known that circPITX1 is a decoy of miR-329-3p, we performed a series of rescue experiments to explore whether miR-329-3p took part in circPITX1-mediated cellular behaviors of glioma cells. U251 and LN229 cells were transfected with si-NC, si-circPITX1, si-circPITX1 + anti-miR-NC or si-circPITX1 + anti-miR-329-3p. At first, qRT-PCR assay disclosed that lack of miR-329-3p overturned si-circPITX1-mediated up-regulation of miR-329-3p (Fig. 4a). The declined cell viability of U251 and LN229 cells caused by si-circPITX1 was obviously recovered by miR-329-3p inhibitors (Fig. 4b, c). Additionally, si-circPITX1-induced reduction of glucose consumption, lactate production and ATP level was relieved by the addition of miR-329-3p inhibitors (Fig. 4d–f). Similarly, miR-329-3p inhibitors also weakened the hindered effect of si-circPITX1 on protein levels of HIF-1α, HK2 and LDH-A (Fig. 4g, h). Colony formation assay manifested that the suppressive effect of circPITX1 knockdown on the survival rate and colony formation rate was attenuated by the cotransfection with miR-329-3p inhibitors under radiation treatment or not (Fig. 4i–l). Taken together, circPITX1 knockdown impeded the glycolysis and radioresistance of glioma cells by up-regulating miR-329-3p.

### MiR-329-3p directly targeted NEK2

**Fig. 5:**
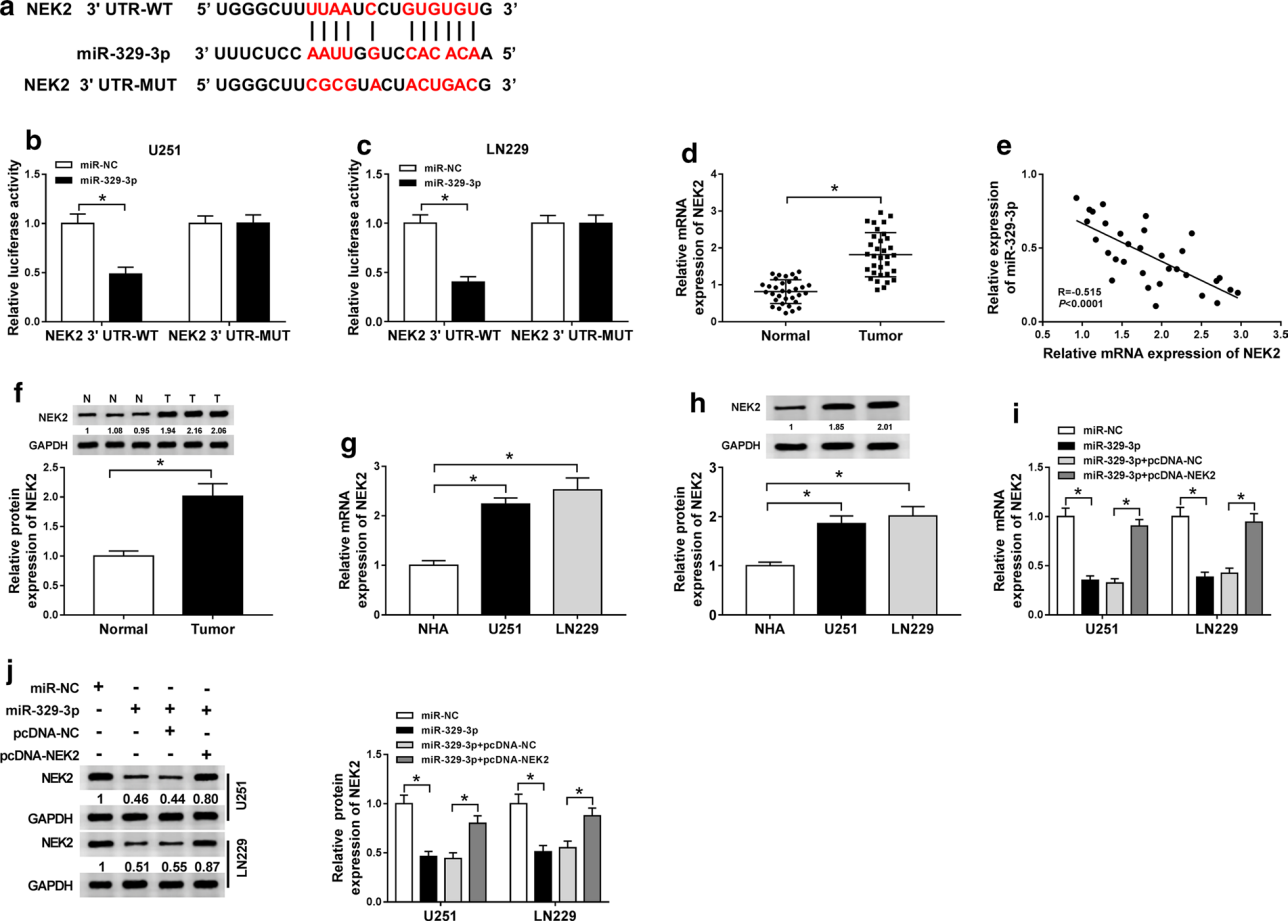
MiR-329-3p directly targeted NEK2. **a** The binding sites between miR-329-3p and NEK2, as well as the mutant. **b**, **c** Dual-luciferase reporter assay for the luciferase activity in glioma U251 and LN229 cells cotransfected with NEK2 3′UTR-WT or NEK2 3′UTR-MUT and miR-329-3p or miR-NC. **d** QRT-PCR assay for the relative mRNA expression of NEK2 in glioma tissues and normal tissues. **e** Pearson correlation analysis for the correlation between the expression of miR-329-3p and NEK2 in glioma tissues. R = − 0.515, *P *< 0.0001. **f** Western blot assay for the protein level of NEK2 in glioma tissues and normal tissues. **g** QRT-PCR assay for the relative mRNA expression of NEK2 in glioma U251 and LN229 cells and NHA. **h** Western blot assay for the protein level of NEK2 in glioma U251 and LN229 cells and NHA. **i** QRT-PCR assay for the relative mRNA expression of NEK2 in U251 and LN229 cells transfected with miR-NC, miR-329-3p, miR-329-3p + pcDNA-NC or miR-329-3p + pcDNA-NEK2. **j** Western blot assay for the protein level of NEK2 in U251 and LN229 cells transfected with miR-NC, miR-329-3p, miR-329-3p + pcDNA-NC or miR-329-3p + pcDNA-NEK2. *P < 0.05

Next, we intended to seek the functional target of miR-329-3p that could help identify the role of miR-329-3p in glioma. The target of miR-329-3p was identified by StarBase 3.0, 3′UTR of NEK2 harbored binding sites to miR-329-3p (Fig. 5a). Furthermore, dual-luciferase reporter assay implied that miR-329-3p triggered about 55% decrease of the luciferase activity in NEK2 3′UTR-WT group rather than NEK2 3′UTR-MUT group in U251 and LN229 cells, suggesting that miR-329-3p targeted NEK2 (Fig. 5b, c). What’s more, we found that NEK2 mRNA was up-regulated in glioma tissues when compared with normal tissues (Fig. 5d), and was negatively correlated with miR-329-3p expression in glioma tissues (Fig. 5e). As expected, NEK2 protein level was also higher in glioma tissues in reference to normal tissues (Fig. 5f). We also identified the up-regulation of NEK2 in glioma U251 and LN229 cells versus NHA, at mRNA and protein levels (Fig. 5g, h). In addition, miR-329-3p could inhibit the mRNA and protein levels of NEK2, while gain of NEK2 abolished the repressed effect (Fig. 5i, j). Thus, we concluded that NEK2 was a downstream gene of miR-329-3p, and was inversely regulated by miR-329-3p.

### Up-regulation of NEK2 reversed the miR-329-3p-induced repressed effects on the glycolysis and radioresistance of glioma cells

**Fig. 6:**
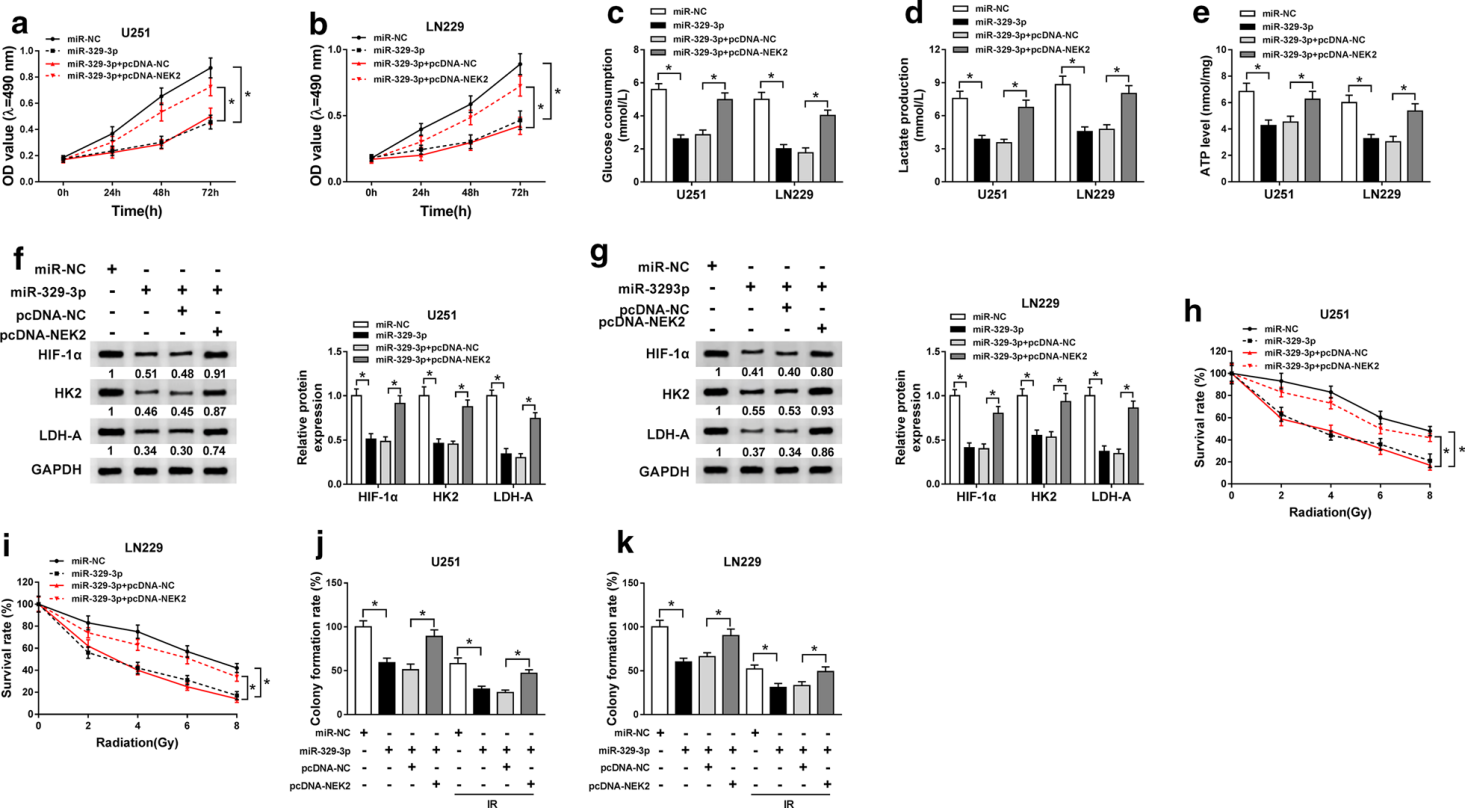
Up-regulation of NEK2 reversed the miR-329-3p-induced repressed effects on the glycolysis and radioresistance of glioma cells. **a**–**g** U251 and LN229 cells were transfected with miR-NC, miR-329-3p, miR-329-3p + pcDNA-NC or miR-329-3p + pcDNA-NEK2. **a**, **b** MTT assay for the viability of transfected glioma cells. **c**–**e** The glucose consumption, lactate production and ATP level in transfected U251 and LN229 cells. **f**, **g** Western blot assay for the levels of HIF-1α, HK2 and LDH-A in transfected U251 and LN229 cells. **h, i** Colony formation assay for the survival rate of U251 and LN229 cells transfected with miR-NC, miR-329-3p, miR-329-3p + pcDNA-NC or miR-329-3p + pcDNA-NEK2 under X-ray radiation treatment (0 Gy, 2 Gy, 4 Gy, 6 Gy and 8 Gy). **j**, **k** Colony formation assay for the colony formation rate of U251 and LN229 cells transfected with miR-NC, miR-329-3p, miR-329-3p + pcDNA-NC or miR-329-3p + pcDNA-NEK2 treated with 6 Gy of X-rays or not. *P < 0.05

To determine the impact of miR-329-3p on the development of glioma and the potential mechanism, rescue experiments were carried out. U251 and LN229 cells were transfected with miR-NC, miR-329-3p, miR-329-3p + pcDNA-NC or miR-329-3p + pcDNA-NEK2. MTT assay uncovered that miR-329-3p evidently retarded cell viability of glioma cells, while overexpression of NEK2 ameliorated the inhibition (Fig. 6a, b). Following glycolysis assay and western blot analysis signified that miR-329-3p-induced glycolysis inhibition was remitted by up-regulation of NEK2 (Fig. 6c–g). The data from colony formation assay indicated that repression of survival rate and colony formation rate triggered by miR-329-3p mimics was weakened by cotransfection with pcDNA-NEK2 in U251 and LN229 cells with or without radiation treatment (Fig. 6h–k). In short, up-regulation of NEK2 could relieve the repressed effects of miR-329-3p on the glycolysis and radioresistance of glioma U251 and LN229 cells.

### CircPITX1 positively regulated NEK2 expression by absorbing miR-329-3p

**Fig. 7:**
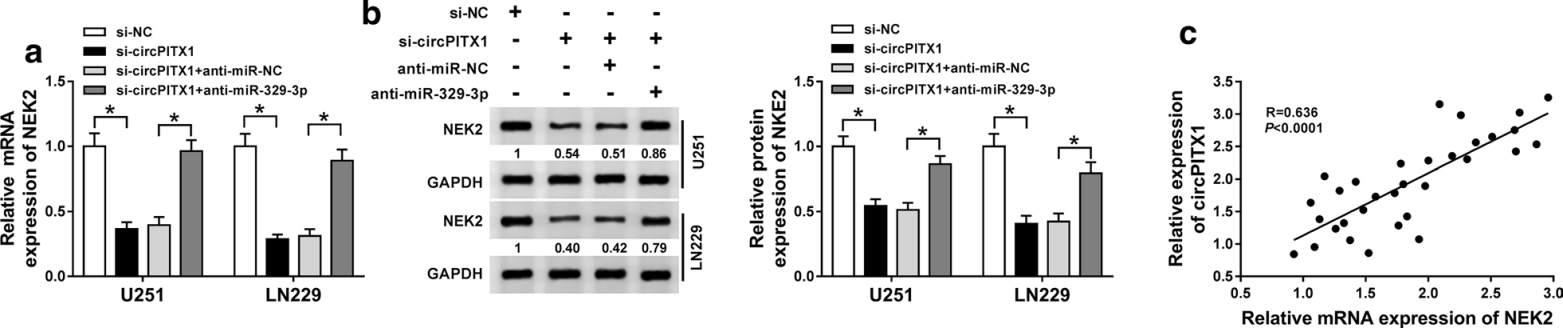
CircPITX1 positively regulated NEK2 expression by absorbing miR-329-3p. **a**, **b** U251 and LN229 cells transfected with si-NC, si-circPITX1, si-circPITX1 + anti-miR-NC or si-circPITX1 + anti-miR-329-3p. **a** QRT-PCR assay for the relative mRNA expression of NEK2 in transfected cells. **b** Western blot assay for the relative protein of NEK2 in transfected cells. **c** Pearson correlation analysis for the correlation between the expression of circPITX1 and NEK2 in glioma tissues. R = 0.636, *P *< 0.0001. *P < 0.05

To confirm the existence of the regulatory axis circPITX1/miR-329-3p/NEK2 in glioma, we tried to clarify the effect of circPITX1 on NEK2 level. U251 and LN229 cells were transfected with si-NC, si-circPITX1, si-circPITX1 + anti-miR-NC or si-circPITX1 + anti-miR-329-3p. qRT-PCR and western blot assays uncovered that circPITX1 knockdown down-regulated NEK2, while recovered by miR-329-3p inhibitors (Fig. 7a, b). A significant positive correlation between the expression of circPITX1 and NEK2 in glioma tissues was confirmed by Pearson correlation analysis (Fig. 7c). To sum up, circPITX1 positively regulated NEK2 level by sponging miR-329-3p.

### Glycolysis inhibitor 2-deoxy-d-glucose (2-DG) treatment weakened the elevated effects of circPITX1 on glycolysis

**Fig. 8:**
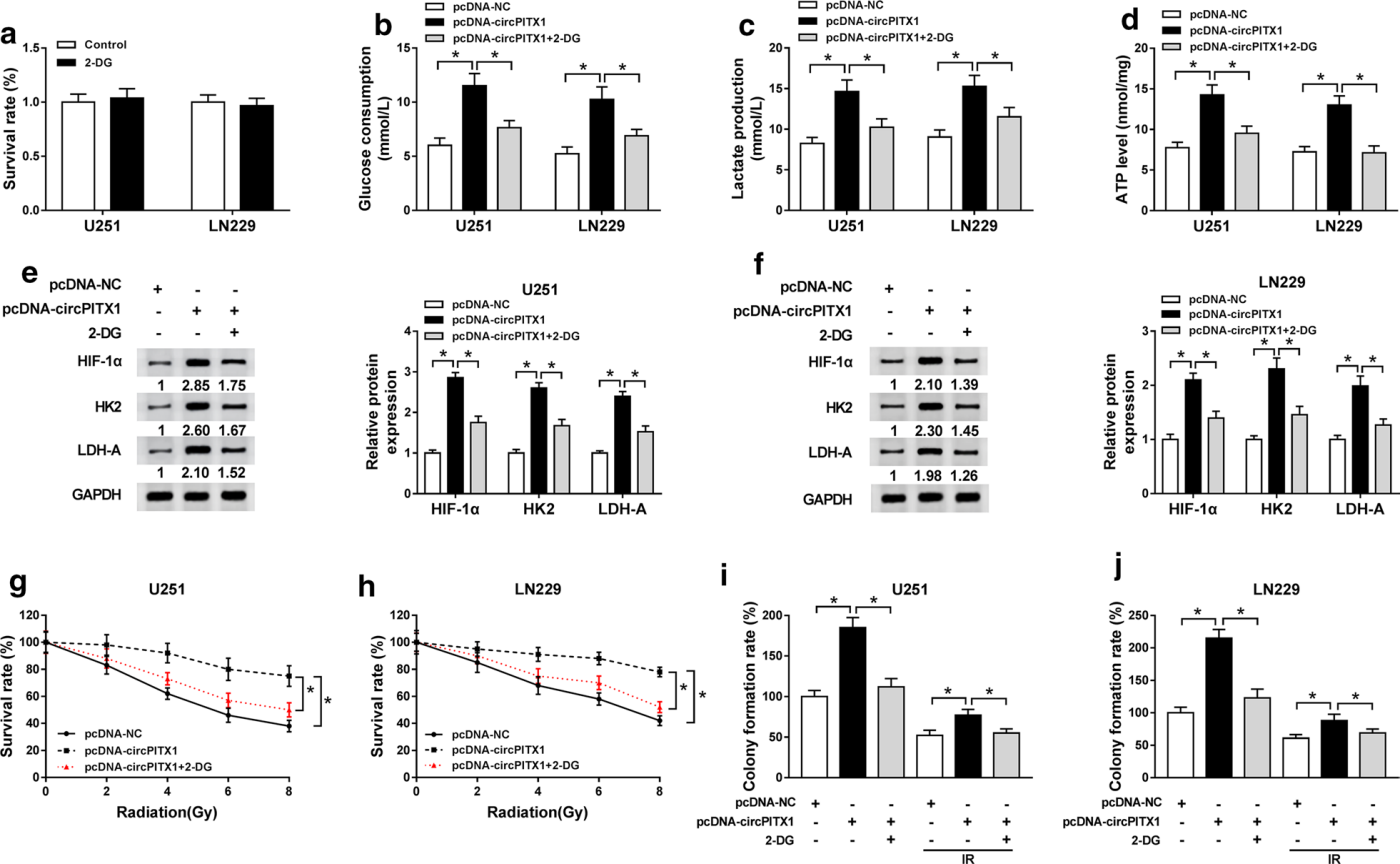
Glycolysis inhibitor 2-deoxy-d-glucose (2-DG) treatment weakened the elevated effects of circPITX1 on glycolysis. **a** Colony formation assay for the survival rate of U251 and LN229 cells treated with 2-DG or not (Control). **b**–**f** U251 and LN229 cells treated with pcDNA-NC, pcDNA-circPITX1 or pcDNA-circPITX1 + 2-DG. **b**–**d** The glucose consumption, lactate production and ATP level in treated U251 and LN229 cells. **e**, **f** Western blot assay for the levels of HIF-1α, HK2 and LDH-A in treated U251 and LN229 cells. **g**, **h** Colony formation assay for the survival rate of U251 and LN229 cells treated with pcDNA-NC, pcDNA-circPITX1 or pcDNA-circPITX1 + 2-DG under X-ray radiation treatment (0 Gy, 2 Gy, 4 Gy, 6 Gy and 8 Gy). **i**, **j** Colony formation assay for the colony formation rate of U251 and LN229 cells treated with pcDNA-NC, pcDNA-circPITX1 or pcDNA-circPITX1 + 2-DG with 6 Gy of X-rays treatment or not. *P < 0.05

To make it clear how the circPITX1 knockdown facilitated the radiosensitivity of glioma cells, 2-DG was introduced to selectively hinder the glycolysis in U251 and LN229 cells [24]. 2-DG management did not affect the survival rate of glioma cells (Fig. 8a). As we can see in Fig. 8b–f, U251 and LN229 cells transfected with pcDNA-circPITX1 under 2-DG treatment no longer displayed high levels of glucose consumption, lactate production and ATP level, as well as up-regulated levels of HIF-1α, HK2 and LDH-A. Colony formation assay suggested that elevation of survival rate and colony formation rate due to circPITX1 up-regulation was counteracted by 2-DG disposition in U251 and LN229 cells with or without radiation treatment (Fig. 8g–j). Therefore, circPITX1-induced glycolysis promotion was undermined by 2-DG. In other words, silencing of circPITX1 impeded glycolysis to elevate radiosensitivity of glioma.

### Silence of circPITX1 inhibited tumor growth of glioma via modulating miR-329-3p/NEK2 axis

**Fig. 9:**
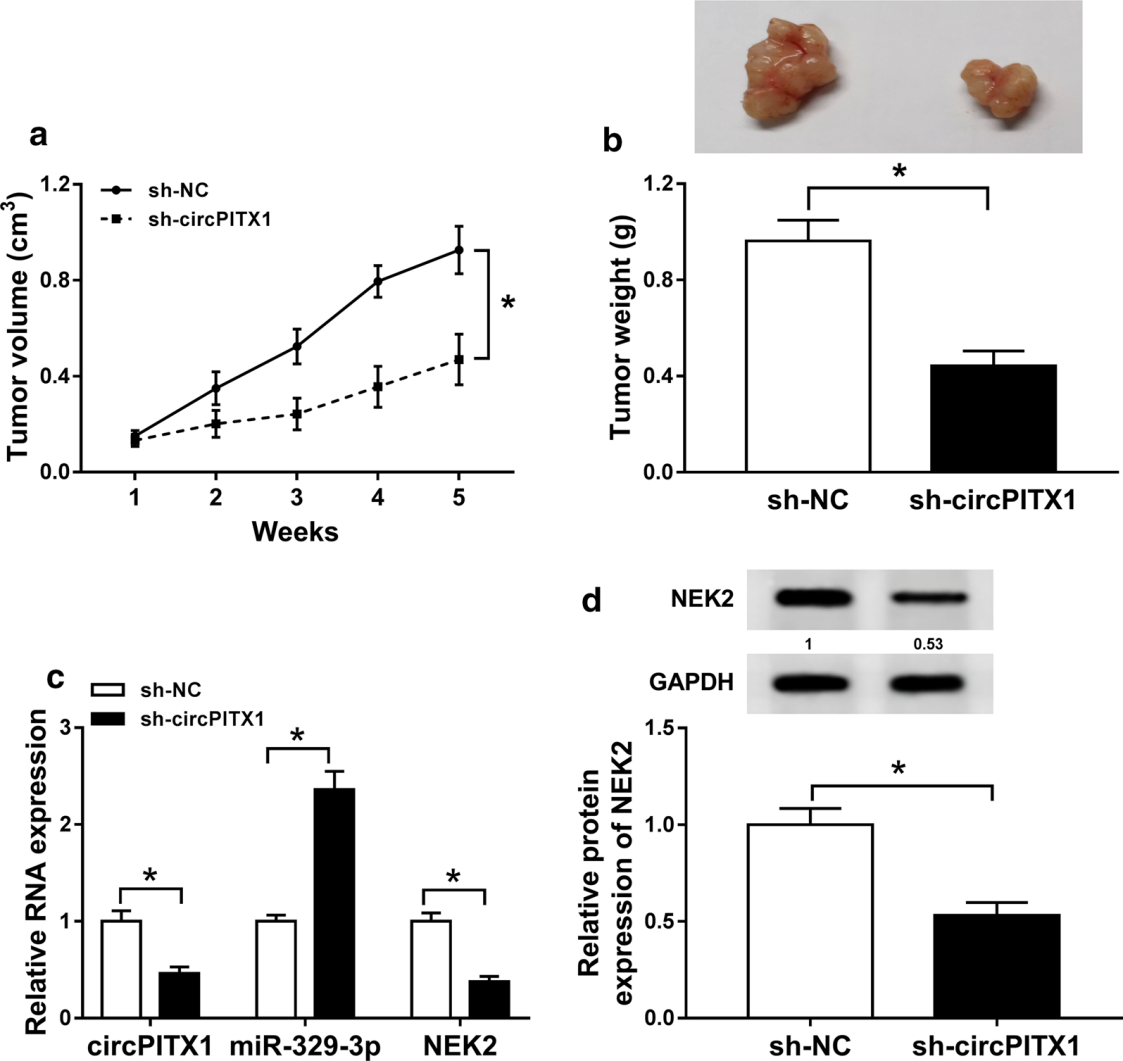
Silencing of circPITX1 inhibited tumor growth of glioma via modulating miR-329-3p/NEK2 axis. BALB/c nude mice were subcutaneously injected with LN229 cells stably expressed sh-circPITX1 or sh-NC. **a** Volume of generated tumors recorded once a week. **b** Photo and weight of generated tumors at 5 weeks post injection. **c** QRT-PCR assay for the relative expression of circPITX1, miR-329-3p and NEK2 in generated tumors. **d** Western blot assay for the protein level of NEK2 in generated tumors. *P < 0.05

Tumor xenograft model was established to explore the role of circPITX1 in vivo. The volume and weight of tumor generated in sh-circPITX1 group were conspicuously lower than that in sh-NC group (Fig. 9a, b). Through further analyzation of expression of circPITX1, miR-329-3p and NEK2, we found that circPITX1 and NEK2 were declined, whereas miR-329-3p was augmented in sh-circPITX1 group in comparison with sh-NC group (Fig. 9c, d). Shortly, deficiency of circPITX1 blocked tumor growth of glioma via miR-329-3p/NEK2 axis.

## Discussion

As the most prevailing primary tumor in head, glioma results in severe mortality, which composes 80% of brain malignancies [25]. Though the incidence of glioma is relatively low, the 5-year survival rate of the most common glioma, glioblastoma, is about 5% [25]. Therefore, deep investigation on glioma evolvement is of urgent need.

Increasing evidence revealed that multiple circRNAs were involved in the regulation of cellular phenotype of glioma cells through diverse pathways, functioning as oncogenes, like circHIPK3 [11], hsa-circ-0014359 [12] and circCPA4 [13]; or playing as tumor suppressors, such as circ-FBXW7 [26], circ-SHPRH [27], and circ_0001946 [28]. In this project, we focused on circPITX1, an oncogene in glioma [15]. When compared to normal tissues and NHA, circPITX1 was upregulated in glioma tissues and glioma U251 and LN229 cells, which was in accordance with the study scheduled by Lv et al. [29]. We further examined the stability of circPITX1. The data proved that circPITX1 was tolerant to RNase R digestion. Following functional assays were executed to analyze the biological role of circPITX1 in glioma progression. We found that deficiency of circPITX1 suppressed glioma cell viability, glycolysis, radioresistance clonogenicity capacity in vitro and tumor growth in vivo, suggesting that circPITX1 was a positive modulator in the development of glioma.

During the investigation of human cancers, researchers observed that connections and interactions between different kinds of RNAs were fairly complicated [30]. According to ceRNA hypothesis, circRNA could inhibit the expression levels of targeted miRNAs, thus affecting the expression and function of mRNAs. For example, circPITX1 sponged miR-1304 to modulate ERBB4 expression, leading to promotion in glioma progression [15]. CircPITX1 facilitated glioblastoma evolvement via acting as a competing endogenous RNA to regulate MAP3K2 by absorbing miR-379-5p [29]. Additionally, introduction of circPITX1 could up-regulate IL17RD abundance via reducing the expression of miR-518a-5p and worsen glioma [31]. Here, in this project, miR-329-3p was predicted by StarBase 3.0 and confirmed by dual-luciferase reporter assay to be a target miRNA of circPITX1.

Xiao et al. verified that miR-329-3p might decrease cell cycle progression in glioma by the targeted inhibition on E2F1, playing a tumor suppressor role [32]. From our data, miR-329-3p expression was declined in glioma samples and glioma U251 and LN229 cells in contrast to corresponding controls, which was in keeping with foregoing reports [19, 32]. Besides, miR-329-3p expression was inversely correlated with circPITX1 in glioma samples. Negatively modulated by circPITX1, miR-329-3p inhibitors could abrogate circPITX1 knockdown-induced repression on glycolysis and radioresistance of glioma cells, signifying that the oncogenic role of circPITX1 was achieved by sponging miR-329-3p.

As we all know, miRNA could bind to the 3′UTR of mRNA to exert diverse functions [33]. In cervical cancer, miR-329-3p directly targeted MAPK1 to retard cell proliferation, migration and invasion of cervical cancer cells [34]. In our study, we proved that NEK2 was a direct target of miR-329-3p. A former study revealed that NEK2 expression was enriched in glioma, and targeted by miR-128 to modulate the apoptosis of glioma cells [35]. Likewise, NEK2 was up-regulated in glioma tissues and cells, at both mRNA and protein levels. Following rescue experiment highlighted the NEK2-mediated abolishment on the decreased glycolysis and radioresistance caused by miR-329-3p. What’s more, circPITX1 positively regulated NEK2 expression by serving as a decoy of miR-329-3p.

In this study, overexpression vector of circPITX1 and 2-DG (glycolysis inhibitor) were simultaneously introduced into glioma U251 and LN229 cells to investigate the circPITX1-induced radioresistance in glioma. Apparently, circPITX1 overexpression facilitated glycolysis process and radioresistance, but 2-DG counteracted the promoted impact, indicating that circPITX1 knockdown inhibited glycolysis to sensitize glioma cells to irradiation treatment.

There still exists certain shortcoming in this study. For instance, HK1, monocarboxylate transporters 1 and 4 (MCT1, MCT4) and glucose transporter isoform 1 (GLUT1) are also glycolysis-related genes [23, 36], which can indicate the glycolytic process as well. And they would be subjected to the investigation of glycolysis in glioma to further monitor the glycolytic process. Besides, in vivo experiments in nude mice were lack of irradiation treatment. It would be better to supplement this treatment into tumor xenograft assay in order to explain the role of circPITX1 in radioresistance of glioma in vivo.

## Conclusion

In general, circPITX1 was highly expressed in glioma and served as an oncogene, exhibiting as the decreased viability, glycolysis, radioresistance, cell survival and clonogenicity capacity, as well as the tumorigenicity inhibition of glioma U251 and LN229 cells triggered by circPITX1 knockdown. Silence of circPITX1 inhibited glycolysis to enhance radiosensitivity in glioma by regulating miR-329-3p/NEK2 axis.

## Data Availability

All data generated or analyzed during this study are included in this published article.

## References

[1] Ricard D, Idbaih A, Ducray F, Lahutte M, Hoang-Xuan K, Delattre JY (2012). Primary brain tumours in adults. Lancet (London, England)..

[2] Zhao J, Liu P, Ma J, Li D, Yang H, Chen W, Jiang Y (2019). Enhancement of radiosensitization by silver nanoparticles functionalized with polyethylene glycol and aptamer As1411 for glioma irradiation therapy. Int J Nanomed.

[3] Epstein T, Gatenby RA, Brown JS (2017). The Warburg effect as an adaptation of cancer cells to rapid fluctuations in energy demand. PLoS ONE.

[4] Ganapathy-Kanniappan S, Geschwind JF (2013). Tumor glycolysis as a target for cancer therapy: progress and prospects. Mol Cancer.

[5] Dong L, He Y, Zhou S, Cao Y, Li Y, Bi Y, Liu G (2019). HIF1alpha-dependent metabolic signals control the differentiation of follicular helper T cells. Cells.

[6] Kudryavtseva AV, Fedorova MS, Zhavoronkov A, Moskalev AA, Zasedatelev AS, Dmitriev AA, Sadritdinova AF, Karpova IY, Nyushko KM, Kalinin DV (2016). Effect of lentivirus-mediated shRNA inactivation of HK1, HK2, and HK3 genes in colorectal cancer and melanoma cells. BMC Genet.

[7] Hou XM, Yuan SQ, Zhao D, Liu XJ, Wu XA (2019). LDH-A promotes malignant behavior via activation of epithelial-to-mesenchymal transition in lung adenocarcinoma. Biosci Rep.

[8] Shen H, Hau E, Joshi S, Dilda PJ, McDonald KL (2015). Sensitization of glioblastoma cells to irradiation by modulating the glucose metabolism. Mol Cancer Ther.

[9] Huang G, Li S, Yang N, Zou Y, Zheng D, Xiao T (2017). Recent progress in circular RNAs in human cancers. Cancer Lett.

[10] Shang Q, Yang Z, Jia R, Ge S (2019). The novel roles of circRNAs in human cancer. Mol Cancer.

[11] Jin P, Huang Y, Zhu P, Zou Y, Shao T, Wang O (2018). CircRNA circHIPK3 serves as a prognostic marker to promote glioma progression by regulating miR-654/IGF2BP3 signaling. Biochem Biophys Res Commun.

[12] Shi F, Shi Z, Zhao Y, Tian J (2019). CircRNA hsa-circ-0014359 promotes glioma progression by regulating miR-153/PI3K signaling. Biochem Biophys Res Commun.

[13] Peng H, Qin C (2019). circCPA4 acts as a prognostic factor and regulates the proliferation and metastasis of glioma. J Cell Mol Med.

[14] Wang R, Zhang S, Chen X, Li N, Li J, Jia R, Pan Y, Liang H (2018). EIF4A3-induced circular RNA MMP9 (circMMP9) acts as a sponge of miR-124 and promotes glioblastoma multiforme cell tumorigenesis. Mol Cancer.

[15] Chen M, Liu X, Xie P, Wang P, Liu M, Zhan Y, Wang H, Feng Y, Li Y (2019). Circular RNA circ_0074026 indicates unfavorable prognosis for patients with glioma and facilitates oncogenesis of tumor cells by targeting miR-1304 to modulate ERBB4 expression. J Cell Physiol.

[16] Wang S, Yin Y, Liu S (2019). Roles of microRNAs during glioma tumorigenesis and progression. Histol Histopathol.

[17] Zhang Y, Chen J (2019). Prognostic significance of MicroRNAs in glioma: a systematic review and meta-analysis. Biomed Res Int.

[18] Chang YH, Yin F, Fan GF, Zhao M (2017). Down-regulation of miR-329-3p is associated with worse prognosis in patients with cervical cancer. Eur Rev Med Pharmacol Sci.

[19] Qiu S, Lin S, Hu D, Feng Y, Tan Y, Peng Y (2013). Interactions of miR-323/miR-326/miR-329 and miR-130a/miR-155/miR-210 as prognostic indicators for clinical outcome of glioblastoma patients. J Transl Med.

[20] Hayward DG, Fry AM (2006). Nek2 kinase in chromosome instability and cancer. Cancer Lett.

[21] Zeng YR, Han ZD, Wang C, Cai C, Huang YQ, Luo HW, Liu ZZ, Zhuo YJ, Dai QS, Zhao HB (2015). Overexpression of NIMA-related kinase 2 is associated with progression and poor prognosis of prostate cancer. BMC Urol.

[22] Liu H, Liu B, Hou X, Pang B, Guo P, Jiang W, Ding Q, Zhang R, Xin T, Guo H (2017). Overexpression of NIMA-related kinase 2 is associated with poor prognoses in malignant glioma. J Neurooncol.

[23] Koch A, Ebert EV, Seitz T, Dietrich P, Berneburg M, Bosserhoff A, Hellerbrand C (2019). Characterization of glycolysis-related gene expression in malignant melanoma. Pathol Res Pract.

[24] Zhang Z, Li TE, Chen M, Xu D, Zhu Y, Hu BY, Lin ZF, Pan JJ, Wang X, Wu C (2019). MFN1-dependent alteration of mitochondrial dynamics drives hepatocellular carcinoma metastasis by glucose metabolic reprogramming. Br J Cancer.

[25] Ostrom QT, Bauchet L, Davis FG, Deltour I, Fisher JL, Langer CE, Pekmezci M, Schwartzbaum JA, Turner MC, Walsh KM (2014). The epidemiology of glioma in adults: a “state of the science” review. Neuro-oncology..

[26] Lu H, Yao B, Wen X, Jia B (2019). FBXW7 circular RNA regulates proliferation, migration and invasion of colorectal carcinoma through NEK2, mTOR, and PTEN signaling pathways in vitro and in vivo. BMC Cancer.

[27] Begum S, Yiu A, Stebbing J, Castellano L (2018). Novel tumour suppressive protein encoded by circular RNA, circ-SHPRH, in glioblastomas. Oncogene.

[28] Li X, Diao H (2019). Circular RNA circ_0001946 acts as a competing endogenous RNA to inhibit glioblastoma progression by modulating miR-671-5p and CDR1. BMC Cancer.

[29] Lv X, Wang M, Qiang J, Guo S (2019). Circular RNA circ-PITX1 promotes the progression of glioblastoma by acting as a competing endogenous RNA to regulate miR-379-5p/MAP3K2 axis. Eur J Pharmacol.

[30] Zhang Y, Li X, Zhou D, Zhi H, Wang P, Gao Y, Guo M, Yue M, Wang Y, Shen W (2018). Inferences of individual drug responses across diverse cancer types using a novel competing endogenous RNA network. Mol Oncol.

[31] Zhan L, Mu Z (2019). Elevation of circ-PITX1 upregulates interleukin 17 receptor D expression via sponging miR-518a-5p and facilitates cell progression in glioma. J Cell Biochem.

[32] Xiao B, Tan L, He B, Liu Z, Xu R (2013). MiRNA-329 targeting E2F1 inhibits cell proliferation in glioma cells. J Transl Med.

[33] Kim D, Chang HR, Baek D (2017). Rules for functional microRNA targeting. BMB Rep.

[34] Li W, Liang J, Zhang Z, Lou H, Zhao L, Xu Y, Ou R (2017). MicroRNA-329-3p targets MAPK1 to suppress cell proliferation, migration and invasion in cervical cancer. Oncol Rep.

[35] Ye Y, Zhi F, Peng Y, Yang CC (2018). MiR-128 promotes the apoptosis of glioma cells via binding to NEK2. Eur Rev Med Pharmacol Sci.

[36] Yao G, Yin J, Wang Q, Dong R, Lu J (2019). Glypican-3 enhances reprogramming of glucose metabolism in liver cancer cells. Biomed Res Int.

